# Modern Imaging and the Future of Prostate Biopsy: Rethinking Systematic Sampling

**DOI:** 10.3390/jimaging12100469

**Published:** 2026-09-26

**Authors:** Georgios Tsakaldimis, Dimitrios Diamantidis, Stavros Lailisidis, Dimitrios Matthaios, Stilianos Giannakopoulos, Christos Kalaitzis

**Affiliations:** 1 Department of Urology, Democritus University of Thrace, 68100 Alexandroupolis, Greecestgian@otenet.gr (S.G.);; 2 Endoscopy Unit, Democritus University of Thrace, 68100 Alexandroupolis, Greece; 3 Department of Medical Oncology, University General Hospital of Alexandroupolis, Faculty of Medicine, Democritus University of Thrace, 68100 Alexandroupolis, Greece

**Keywords:** prostate cancer, prostate biopsy, multiparametric MRI, MRI-targeted biopsy, systematic biopsy, perilesional biopsy, micro-ultrasound, PSMA PET, artificial intelligence

## Abstract

Prostate biopsy has shifted from systematic, non-targeted sampling towards imaging-directed approaches, but the extent to which systematic cores can be safely reduced remains uncertain. This review examines current evidence on contemporary biopsy strategies in men with suspected prostate cancer. A structured PubMed/MEDLINE search through July 2026 identified studies evaluating multiparametric magnetic resonance imaging (mpMRI), MRI-targeted biopsy, perilesional, regional and ipsilateral sampling, micro-ultrasound, prostate-specific membrane antigen positron emission tomography (PSMA PET), radiomics, and artificial intelligence. MRI-targeted biopsy improves the detection of clinically significant disease and reduces low-grade cancer diagnosis, yet targeted sampling alone may miss or undergrade clinically important tumours. Available evidence suggests that perilesional, regional, or ipsilateral sampling can preserve much of the diagnostic contribution of systematic biopsy while reducing unnecessary cores, although the evidence remains heterogeneous and is frequently retrospective. Micro-ultrasound and PSMA PET may further refine lesion localisation, while artificial intelligence may support risk assessment and biopsy planning. Current evidence supports selective reduction in systematic sampling, not its universal omission. Future biopsy strategies should integrate clinical risk, imaging quality, lesion characteristics, and targeting confidence and require prospective multicentre validation before broad implementation.

## 1. Introduction

Prostate biopsy remains the definitive test for the diagnosis of prostate cancer, although the optimal approach to tissue sampling continues to evolve. For many years, systematic biopsy represented the gold standard diagnostic method, providing broad gland coverage through non-targeted sampling. However, this approach requires multiple cores, increases the detection of low-grade disease, and may still fail to identify clinically significant tumours.

The introduction of multiparametric magnetic resonance imaging (mpMRI) substantially changed this diagnostic pathway by allowing suspicious lesions to be visualised before biopsy. MRI-targeted biopsy improves the detection of clinically significant prostate cancer while reducing the diagnosis of clinically insignificant disease compared with systematic biopsy alone [1,2,3]. These findings established mpMRI as a central component of contemporary biopsy planning. Nevertheless, targeted biopsy is not sufficient in every patient. MRI-invisible tumours, variation in image interpretation, targeting error, and multifocal disease may result in clinically significant cancer being missed or underestimated, which explains why systematic cores can still provide additional diagnostic information [4,5].

The question is no longer whether systematic sampling has any value, but whether a complete bilateral template is necessary in every patient with a positive MRI. Full systematic biopsy increases the number of cores and may add disproportionately to the detection of Grade Group 1 disease. This has led to growing interest in more selective approaches, including perilesional, regional, and ipsilateral sampling, which aim to retain the diagnostic benefit of additional cores while avoiding low-yield sampling elsewhere in the gland. At the same time, micro-ultrasound, prostate-specific membrane antigen positron emission tomography, radiomics, and artificial intelligence are providing additional tools for lesion localisation and risk assessment.

Recent reviews have described the broader development of prostate cancer imaging and artificial intelligence [6,7], but less attention has been paid to how these advances should influence the number and spatial distribution of biopsy cores. The present review focuses on the relationship between modern imaging and biopsy strategy, particularly the possibility of reducing systematic sampling without compromising detection of clinically significant cancer.

## 2. Literature Search and Study Selection

A structured literature search was performed in PubMed/MEDLINE to identify studies relevant to contemporary imaging-guided prostate biopsy and reduced systematic sampling. The search included combinations of terms related to “prostate cancer”, “prostate biopsy”, “multiparametric MRI”, “MRI-targeted biopsy”, “systematic biopsy”, “perilesional biopsy”, “regional biopsy”, “ipsilateral biopsy”, “micro-ultrasound”, “PSMA PET”, “radiomics”, and “artificial intelligence”. No lower publication-date limit was applied, and studies indexed up to 12 July 2026 were considered. Reference lists of relevant reviews, meta-analyses, guidelines, and key prospective studies were also checked for additional publications.

Randomised trials, prospective and multicentre studies, systematic reviews, meta-analyses, and large comparative cohorts were prioritised. Studies were included when they provided data on clinically significant prostate cancer detection, the additional diagnostic contribution of systematic sampling, reduced-core biopsy strategies, or emerging imaging modalities used for biopsy guidance or patient selection. Studies dealing exclusively with biochemical recurrence, treatment response, or imaging applications unrelated to primary diagnosis and biopsy planning were excluded. Studies of primary staging were considered only when they also contained information relevant to initial diagnosis, lesion localisation, or biopsy guidance. Conference abstracts, editorials, non-peer-reviewed reports, and studies with insufficient information on biopsy strategy or clinically significant cancer detection were not included. Study identification and selection were performed collaboratively by the authors involved in the literature search (G.T., D.D., and S.L.), with final inclusion decisions reached by consensus. Data extraction was not performed independently in duplicate.

This comprehensive review used a structured narrative approach. This approach was chosen because the review covers a broad and methodologically diverse field, including multiple imaging modalities, biopsy techniques, sampling strategies, and emerging technologies, rather than a single intervention or diagnostic comparison. The aim was to critically synthesise the available evidence and highlight areas of agreement, uncertainty, and future research. Given this scope, the review did not follow a formal systematic-review protocol and did not include a PRISMA flow diagram or a formal study-level risk-of-bias assessment.

ClinicalTrials.gov was searched using the terms “prostate biopsy”, “perilesional biopsy”, “regional biopsy”, “PSMA PET” and “artificial intelligence”, restricted to interventional and diagnostic studies with a recruiting, active or not-yet-recruiting status, in order to identify ongoing work relevant to biopsy extent. Unless otherwise stated, clinically significant prostate cancer denotes International Society of Urological Pathology Grade Group ≥ 2. Where a cited study applied a different threshold, this is indicated in the text or in the corresponding table.

## 3. mpMRI as the Foundation of Contemporary Prostate Biopsy

Multiparametric magnetic resonance imaging (mpMRI) has become central to the diagnostic assessment of men with suspected prostate cancer. Its main contribution is not merely the visualisation of suspicious lesions, but the introduction of a triage step before tissue sampling. In the conventional pathway, an elevated prostate-specific antigen level or an abnormal digital rectal examination led directly to systematic biopsy. With an MRI-directed pathway, the decision to biopsy can be refined based on imaging results and the patient’s clinical risk, while suspicious areas can be sampled directly.

The PROMIS study established the diagnostic role of mpMRI as a pre-biopsy triage test. Among 576 men who underwent mpMRI followed by both transrectal ultrasound-guided and template mapping biopsy, mpMRI reached a sensitivity of 93% and a negative predictive value of 89%. Significant disease in PROMIS was defined as Gleason ≥ 4 + 3 or a maximum cancer core length ≥ 6 mm, a broader threshold than the Grade Group ≥ 2 criterion used in most later work, so these figures are not directly comparable with those quoted below. The investigators estimated that mpMRI used as an initial triage test could allow about 27% of men to avoid immediate biopsy while reducing detection of clinically insignificant disease [8].

Evidence from biopsy-naïve populations established the MRI-directed pathway. In PRECISION, men underwent targeted biopsy only when MRI was suspicious; 28% avoided biopsy, while detection of clinically significant cancer increased and detection of clinically insignificant disease decreased relative to standard systematic biopsy [1]. STHLM3-MRI confirmed that MRI-directed screening maintained clinically significant cancer detection while reducing low-risk diagnoses [9], and a related risk-prediction trial showed that MRI can be incorporated into a sequence of selective tests rather than applied after every elevated PSA result [10]. Similar reductions in unnecessary biopsies and low-grade cancer were observed in a large multicentre cohort [11] and a phase III randomised trial [12]. Meta-analyses confirmed greater detection of clinically significant cancer and less Grade Group 1 disease than with systematic biopsy alone [2,3]; however, a Cochrane review emphasised that some clinically significant cancers may still be missed [13].

mpMRI is not, however, an infallible exclusion or localisation test. Its performance is influenced by acquisition quality, scanner characteristics, adherence to technical standards, and radiologist experience. The PI-RADS Steering Committee has emphasised that the diagnostic benefits of an MRI-directed pathway depend on standardised image acquisition, interpretation, reporting, and integration of MRI findings into the biopsy pathway [14]. Some significant tumours remain inconspicuous, particularly when they are small or located in areas where benign tissue changes complicate interpretation. A visible lesion may also be inadequately sampled because of registration error, prostate deformation, needle displacement, or operator-related limitations. Paired biopsy studies, including MRI-FIRST, have shown that targeted and systematic cores detect overlapping but not identical groups of clinically significant cancers [4]. This residual discordance explains why a negative MRI cannot be interpreted independently of PSA density, digital rectal examination, family history, and the overall probability of cancer.

**Figure 1:**
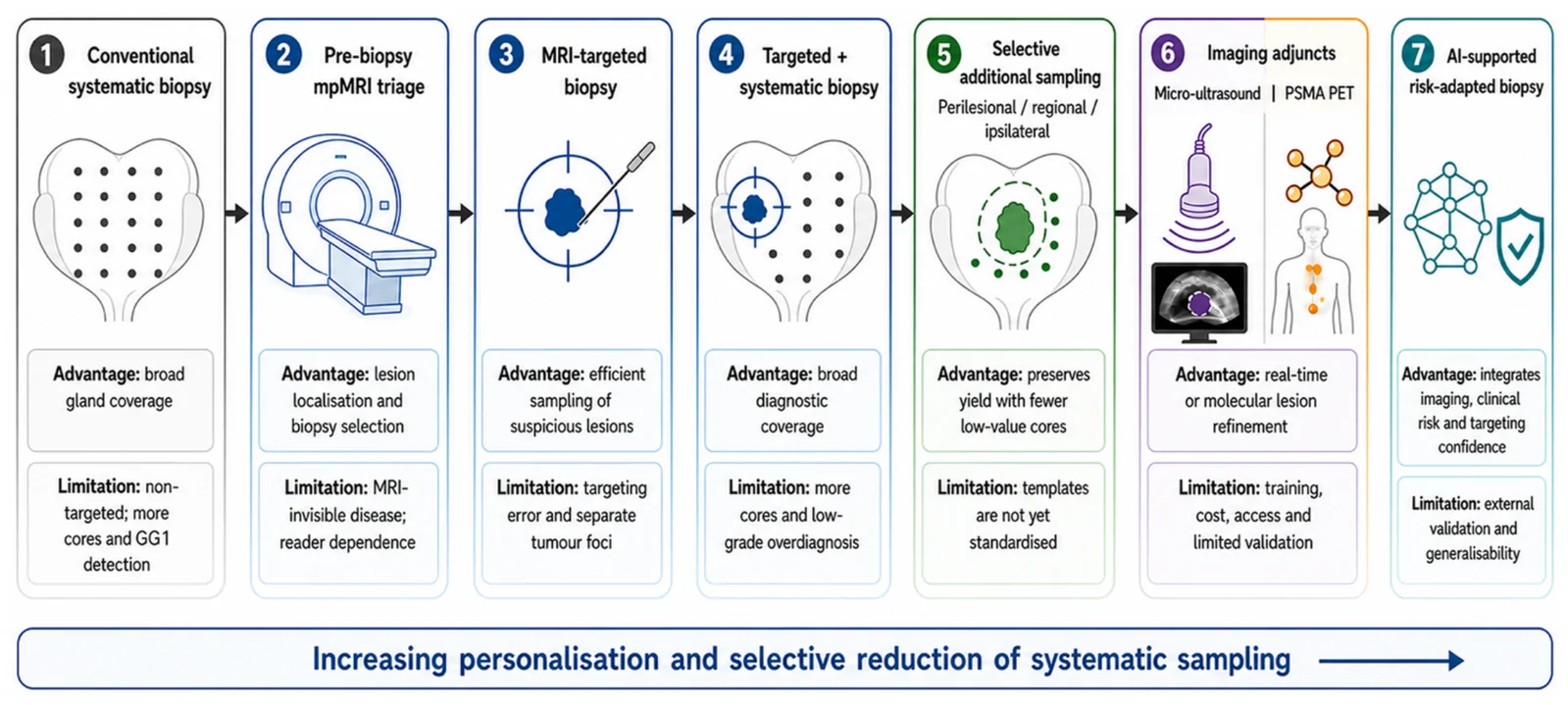
Evolution of prostate biopsy strategies in the imaging era. The figure illustrates the conceptual transition from gland-wide systematic sampling to MRI-directed and increasingly individualised biopsy. Targeted biopsy improves lesion-directed sampling, while perilesional, regional, or ipsilateral cores aim to retain clinically significant cancer detection with fewer low-yield cores. Micro-ultrasound, PSMA PET, and AI are emerging adjuncts rather than universally validated replacements for MRI-based pathways. This is a conceptual framework, not a validated clinical algorithm. Abbreviations: AI, artificial intelligence; GG1, Grade Group 1; mpMRI, multiparametric magnetic resonance imaging; MRI, magnetic resonance imaging; PET, positron emission tomography; PSMA, prostate-specific membrane antigen.

Current guidelines position mpMRI as the basis of modern biopsy planning, but not as a universal justification for omitting additional sampling. The European Association of Urology recommends MRI before biopsy and favours lesion-centred targeted and perilesional sampling in men with positive imaging, while allowing biopsy deferral after negative or equivocal MRI only when clinical suspicion is low [15]. Thus, mpMRI has established the framework for a more selective diagnostic pathway; the remaining question is how far systematic sampling can be reduced without compromising detection of clinically significant disease. The evolution of prostate biopsy strategies from conventional systematic sampling to contemporary imaging-directed and risk-adapted approaches is summarised in Figure 1.

## 4. MRI-Targeted Biopsy and the Residual Role of Systematic Sampling

MRI-targeted biopsy has changed the diagnostic balance in prostate cancer by directing tissue sampling towards lesions most likely to harbour clinically significant disease. In contrast to systematic biopsy, which samples the gland according to a predetermined template, targeted biopsy links imaging findings to histological confirmation. This improves sampling efficiency and reduces the number of cores obtained from MRI-normal tissue. The clinical relevance of this change was demonstrated in PRECISION, in which an MRI-directed pathway detected more clinically significant prostate cancer than standard systematic biopsy while reducing the diagnosis of clinically insignificant tumours [1]. A subsequent meta-analysis of 14,709 men similarly found that MRI-targeted biopsy detected more clinically significant and fewer clinically insignificant cancers than systematic biopsy [2].

These findings, however, do not establish that targeted biopsy is sufficient in every patient. MRI-visible lesions account for most, but not all, clinically significant cancers, and a technically successful biopsy depends on several sequential steps. The lesion must first be visible and correctly interpreted on MRI; its position must then be transferred accurately to ultrasound or the in-bore biopsy environment; finally, the needle must traverse the biologically relevant part of the lesion. Errors at any stage may produce a false-negative or undergraded result. Lesion size, anatomical location, prostate deformation, operator experience, targeting method, and the number of targeted cores may all influence diagnostic yield. Mechanical and robotic systems can improve the reproducibility of needle placement, but they do not eliminate all sources of targeting error. Differences between MRI and biopsy positioning, changes in prostate shape, patient movement, and needle deflection may still alter the final needle trajectory [16]. Moreover, prostate cancer is frequently multifocal, and a dominant MRI lesion does not exclude a separate significant focus elsewhere in the gland.

MRI-FIRST illustrates the complementary yield of targeted and systematic sampling. Among 251 biopsy-naïve men, clinically significant cancer was identified in 94: 13 cancers were detected only by systematic biopsy, 19 only by targeted biopsy, and 62 by both methods [4]. Omitting systematic cores would have missed clinically significant disease in 5.2% of the entire cohort. PAIREDCAP showed a similar pattern in 248 biopsy-naïve men with MRI-visible lesions: targeted and systematic approaches often identified different tumour foci, and their combination achieved the highest detection of clinically significant cancer [17]. These studies show that the two approaches are complementary rather than interchangeable.

Prostatectomy-correlated data provide a second rationale for additional sampling. Combined biopsy showed the closest agreement with whole-mount pathology: upgrading to Grade Group 3 or higher at surgery occurred in 3.5% of men assessed by combined biopsy, compared with 8.7% after targeted biopsy alone and 16.8% after systematic biopsy alone [5]. Additional systematic cores may improve grade assessment as well as cancer detection, with implications for surveillance, focal treatment, radical therapy, and staging.

The residual benefit of systematic biopsy must nevertheless be weighed against its disadvantages. A full bilateral template adds cores and procedural burden and exposes a larger volume of prostatic tissue to needle sampling. It also detects additional Grade Group 1 disease, potentially leading to anxiety, repeated surveillance procedures, and overtreatment. The diagnostic gain from systematic biopsy is not uniformly beneficial: identification of an MRI-occult Grade Group 2 tumour may alter management, whereas detection of a small low-grade focus may add burden without offering a comparable oncological benefit. Meta-analytic evidence confirms the greater selectivity of MRI-targeted biopsy for clinically significant disease and its lower detection of clinically insignificant cancer [2,3]. Recent randomised evidence indicates that the systematic component of combined biopsy can be reduced without being abandoned. In the SEXTANT-PRO non-inferiority trial, 506 biopsy-naïve men with a single MRI-visible lesion were randomised to undergo MRI-targeted biopsy combined with either six-core sextant systematic sampling or conventional 12-core systematic biopsy. Clinically significant prostate cancer was detected in 138 of 254 men (54.3%) with sextant sampling and in 138 of 252 (54.8%) with the 12-core template (difference −0.5%, 95% confidence interval −9.2% to 8.3%), meeting the prespecified non-inferiority margin of −15%. That margin is wide for a diagnostic question with oncological consequences, and its lower limit still admits a loss of approximately nine percentage points in clinically significant cancer detection. The trial provides randomised evidence that, in biopsy-naïve men with a solitary MRI-visible lesion, halving the systematic template preserves detection while reducing tissue sampling [18].

The balance between diagnostic protection and unnecessary sampling is also unlikely to be identical across all patients. Men undergoing their first biopsy, those assessed with lower-quality or externally performed MRI, patients with large or anatomically challenging glands, and cases in which targeting confidence is limited may derive greater benefit from broader sampling than men with a highly conspicuous lesion evaluated in an experienced centre. Such heterogeneity limits the validity of a uniform targeted-only strategy and explains why the residual role of systematic biopsy remains clinically relevant.

The unresolved question is whether the residual value of systematic sampling requires a complete bilateral template in every MRI-positive patient. In selected cohorts, most additional clinically significant disease is detected on the side of the MRI-visible lesion, whereas exclusively contralateral findings contribute relatively little [19]. This observation underpins perilesional, regional, and ipsilateral strategies designed to retain diagnostic protection while reducing low-yield cores and Grade Group 1 detection.

## 5. Perilesional, Regional, and Ipsilateral Biopsy Strategies

Spatial uncertainty around an MRI-visible lesion provides the rationale for limited additional sampling. The MRI contour may not correspond exactly to the histological tumour boundary, and registration error, prostate deformation, needle deflection, or operator-related error may divert a targeted core into adjacent tissue. Additional cores near the target can compensate for these limitations without routinely sampling the entire MRI-negative gland. Perilesional, regional, and ipsilateral strategies are different practical expressions of this principle.

Terminology remains inconsistent. Perilesional biopsy generally refers to cores taken immediately around the MRI target, regional biopsy includes the target and adjacent sectors, and ipsilateral biopsy retains systematic sampling from the involved lobe while omitting the contralateral lobe. These approaches also differ technically: a narrow perilesional template requires accurate segmentation and reliable fusion or in-bore guidance, whereas ipsilateral sampling is easier to reproduce during cognitive targeting. The recurrent 10 mm margin is operationally convenient but biologically arbitrary: it assumes reproducible lesion boundaries and needle placement and is unlikely to transfer unchanged across fusion, in-bore, and cognitive workflows. All three strategies aim to recover clinically significant cancer missed by targeted cores while reducing low-yield sampling elsewhere.

Spatial mapping provides the biological rationale for this approach. Spatial mapping by Brisbane and colleagues described the visible lesion as the tumour “umbra” and the adjacent area as its “penumbra”; 90% of cancer-positive cores lay within 10 mm of the nearest MRI lesion [20]. Because this was a core-level analysis, the estimate should not be transferred directly to patient-level decisions. Noujeim and colleagues subsequently reported that a 10 mm perilesional strategy detected clinically significant cancer in 35% of patients, compared with 37% using targeted plus full systematic biopsy [21]. Disease beyond the margin was more frequent in patients with adverse risk features, arguing against a fixed distance for every case.

Ipsilateral-sampling cohorts provide complementary evidence. Bryk and colleagues reported 96% sensitivity for Grade Group 2 or higher disease when MRI-targeted biopsy was combined with ipsilateral systematic cores, with less detection of insignificant cancer than with bilateral systematic sampling [22]. In the Freifeld cohort, sensitivity increased from 85.5% with targeted biopsy alone to 96.4% after ipsilateral systematic cores were added [23]. These results position ipsilateral or regional sampling between targeted-only biopsy and a full bilateral template.

Later cohorts reported a similar pattern. In the Jager cohort of men with a unilateral MRI lesion, targeted plus ipsilateral systematic biopsy retained 98.6% sensitivity for clinically significant disease, missing 1.4% of such cancers and detecting fewer insignificant cancers [24]. Ruan and colleagues estimated in a larger reconstructed cohort that omitting contralateral cores would have missed only 0.3% of clinically significant cancers identified by the full combined approach [25]. In the cognitive-targeting study by Yu and colleagues, targeted plus ipsilateral sampling detected 95.9% of clinically significant cancers [26]. Because fusion platforms are not universally available, strategies dependent on software-defined margins may be difficult to reproduce in community or resource-limited settings.

Direct protocol studies address an important limitation of retrospective reconstructions of a full biopsy. In the Hagens cohort, targeted plus perilesional cores detected 96.8% of the clinically significant cancers found by the full combined strategy, reduced the mean core count by 5.2, and avoided some systematic-only Grade Group 1 diagnoses [27]. The prospective, non-randomised, surgeon-blinded Duwe study was less supportive: targeted plus perilesional biopsy did not preserve clinically significant cancer detection to the same extent as targeted plus full systematic biopsy [28]. Although smaller than several reconstructed cohorts, the Duwe study carries disproportionate evidential weight because it tested an intentionally limited procedure prospectively. Retrospective reconstruction from a complete biopsy assumes that the retained cores would have been placed identically had the operator known that no further cores would be taken. The randomised Jiang trial of 434 biopsy-naïve men evaluated regional saturation around the MRI-defined lesion and found clinically significant cancer detection comparable to combined targeted and systematic biopsy (44.1% vs. 40.7%) with fewer cores [29]. These data support a regional safety margin but do not define a universally optimal number, distance, or distribution of cores.

Pooled analyses place regional biopsy between targeted-only and full systematic sampling. A meta-analysis of eight studies including 2603 MRI-positive men found no statistically significant difference in Grade Group 2 or higher detection between targeted plus regional biopsy and targeted plus full systematic biopsy. Regional sampling, however, detected significantly more clinically significant cancer than targeted biopsy alone [30]. A later meta-analysis of 21 studies similarly reported clinically significant cancer detection rates of 44.2% with regional sampling and 46.1% with full systematic sampling, while the full approach diagnosed more Grade Group 1 disease [31]. These findings support selective reduction in background sampling, not its removal.

A network meta-analysis comparing 13 biopsy strategies in MRI-positive patients found that targeted biopsy plus ipsilateral systematic sampling achieved clinically significant cancer detection comparable to targeted biopsy plus full systematic sampling, whereas targeted biopsy alone provided less diagnostic protection [32]. This synthesis supports selective reduction rather than complete omission of systematic sampling.

This interpretation is reflected in current European Association of Urology guidance. For a positive MRI, targeted biopsy should be combined with perilesional sampling for PI-RADS 4–5 lesions; in PI-RADS 3 lesions, biopsy decisions should also incorporate clinical suspicion, particularly PSA density and family history. The recommendation remains weak because the number and spatial distribution of perilesional cores have not been standardised, and the underlying evidence is largely observational [15].

Guideline positions are not uniform. The EAU recommends targeted biopsy with perilesional sampling for PI-RADS 4–5 lesions and risk-adapted decisions for PI-RADS 3. The AUA/SUO early detection guideline recommends targeted biopsy in biopsy-naïve men with a suspicious MRI lesion and allows systematic template biopsy as a conditional option [33], without endorsing a perilesional template as a substitute. European guidance explicitly supports perilesional sampling, whereas North American guidance is less prescriptive and generally retains systematic sampling as an option. Consider this difference when applying reduced-template data across settings. Table 1 summarises the principal studies.

Several limitations prevent immediate adoption of one reduced template for all patients. Many studies retrospectively reclassified cores obtained during a full combined biopsy, creating a hypothetical limited strategy instead of testing one prospectively. Definitions of “perilesional,” “regional,” and “ipsilateral” vary, as do biopsy route, targeting method, previous biopsy status, lesion number, and reference standard. Whole-mount prostatectomy correlation is uncommon, and small MRI-occult tumours in the contralateral lobe may be underestimated. Broader sampling remains appropriate when MRI quality is uncertain, targeting confidence is low, the gland is large or anatomically difficult, multiple lesions are present, or clinical risk appears disproportionate to the imaging findings. Biopsy route is another source of heterogeneity. With transperineal access, the needle can be directed from the apical region towards the base, facilitating sampling of anterior and apical areas. In contrast, transrectal biopsy approaches the gland from the posterior surface with a more oblique needle trajectory, which may make these regions more difficult to sample. These geometric differences may also influence targeted and perilesional biopsy, as the same number of cores may not provide equivalent anatomical coverage with the two routes. In the PREVENT randomised trial of 658 men, infectious complications occurred in no patient after transperineal biopsy and in four (1.4%) after transrectal biopsy, while clinically significant cancer detection was similar (53% vs. 50%; adjusted difference 2.0%, 95% confidence interval −6.0 to 10) [34]. Reduced-sampling studies used different routes: SEXTANT-PRO, the principal perilesional cohort, and PRIMARY2 used transperineal access, whereas the main ipsilateral cohorts used transrectal access. A template validated with one route cannot be assumed to transfer directly to the other. Future trials should stratify by biopsy access.

The emerging evidence supports a move away from the binary choice between targeted biopsy alone and a full bilateral systematic template. When accurate spatial targeting is available, additional cores concentrated around the lesion may compensate for segmentation and needle-placement error. When such precision is not feasible, ipsilateral systematic sampling provides a pragmatic alternative. The objective is not core minimisation. It is the removal of cores with low diagnostic value, with detection and grading of clinically significant cancer preserved.

## 6. Micro-Ultrasound

Micro-ultrasound (microUS) has emerged as a real-time imaging method for prostate cancer detection and biopsy guidance. Unlike conventional transrectal ultrasound, which mainly provides anatomical orientation, microUS operates at 29 MHz and offers substantially improved spatial resolution. This allows subtle alterations in glandular and ductal architecture to be assessed during the biopsy itself. Suspicious regions are classified using the Prostate Risk Identification using a Micro-Ultrasound (PRI-MUS) system, a five-point scale in which scores of 3–5 indicate increasing concern for clinically significant prostate cancer. Because lesion identification and needle placement occur on the same platform, microUS avoids the registration step required for MRI–ultrasound fusion [35,36].

The clinical appeal of microUS is a single-session workflow that does not require preprocedural segmentation, dedicated fusion software, or MRI access during biopsy. The operator can survey the gland, identify suspicious areas, direct targeted cores, and add background sampling, which may be useful where MRI capacity is limited. MicroUS is not simply a higher-resolution version of conventional ultrasound: interpretation requires specific training, and inflammation, atrophy, and benign prostatic hyperplasia can mimic cancer [35,36].

Early prospective studies suggested that microUS could detect clinically significant disease at rates comparable to mpMRI. A multicentre prospective registry reported similar diagnostic performance for the two modalities, with high sensitivity for Grade Group 2 or higher cancer using microUS [37]. Meta-analyses found no clear difference between microUS-targeted and MRI-targeted biopsy, although most included studies were observational and varied in protocol, blinding, operator experience, and use of systematic cores [38]. Available comparisons with systematic biopsy did not justify routine omission of background sampling [39].

OPTIMUM provides the strongest comparative evidence. Of 802 randomised biopsy-naïve men, 678 underwent biopsy. In the primary comparison, Grade Group 2 or higher cancer was detected in 47.1% of 121 men assigned to microUS and 42.6% of 331 assigned to MRI-ultrasound fusion (difference 3.52%, 95% confidence interval −3.95% to 10.92%), within the prespecified 10% non-inferiority margin. Because systematic biopsy was performed in every arm, the trial compares microUS plus systematic sampling with MRI plus systematic sampling and does not test omission of background cores [40]. The findings support microUS as a credible image-guided platform but do not establish superiority to mpMRI or make pre-biopsy MRI unnecessary.

The two modalities provide different types of information. Multiparametric MRI offers multiplanar assessment of the entire gland, facilitates evaluation of anterior and apical lesions, and contributes to local staging and treatment planning. MicroUS provides dynamic, high-resolution imaging during needle placement and allows immediate adjustment of the biopsy trajectory. Their findings are often complementary. A lesion visible on one modality may be inconspicuous on the other, and combined use may increase sensitivity at the cost of more targets, additional cores, and more false-positive findings.

Several barriers remain before microUS can be incorporated into routine pathways. PRI-MUS was initially developed for the peripheral zone, while interpretation of anterior and transition-zone abnormalities continues to evolve. Evidence is also limited regarding reproducibility, negative predictive value in biopsy avoidance, cost-effectiveness, and outcomes after a negative examination. Furthermore, systematic sampling was retained in many studies, making it difficult to determine whether microUS alone can safely replace broader biopsy templates.

MicroUS can integrate lesion detection and targeted sampling in real time and may serve as an alternative or complement to MRI guidance. Its ability to reduce systematic cores remains unproven and requires standardised training, external validation, and prospective reduced-sampling trials.

## 7. PSMA PET in Primary Prostate Cancer Diagnosis and Biopsy Guidance

Prostate-specific membrane antigen positron emission tomography (PSMA PET) is established primarily for staging and recurrence imaging, but its role in the initial diagnosis of prostate cancer is increasingly being investigated. PSMA is a transmembrane protein expressed at higher levels in most prostate cancers than in benign prostatic tissue, with expression generally increasing in biologically aggressive disease. Radiolabelled PSMA ligands provide molecular information that differs from the anatomical and functional information obtained with multiparametric magnetic resonance imaging (mpMRI). This distinction has raised interest in PSMA PET as a complementary method for identifying clinically significant prostate cancer and refining biopsy selection.

The strongest early prospective evidence came from the PRIMARY trial. In this multicentre study, 291 biopsy-naïve men underwent mpMRI, ^68Ga-PSMA PET/CT, and transperineal biopsy. The addition of PSMA PET to mpMRI increased sensitivity for clinically significant prostate cancer from 83% to 97% and improved the negative predictive value from 72% to 91% [41]. The benefit was particularly relevant in men with negative or equivocal MRI findings, in whom molecular imaging identified additional disease not adequately characterised by mpMRI alone. These results suggest that PSMA PET may function more effectively as an adjunct to MRI than as a direct replacement.

The PRIMARY Score was subsequently developed to standardise intraprostatic PSMA PET interpretation. It classifies findings according to uptake pattern, anatomical distribution, and intensity, ranging from no abnormal uptake to intense focal uptake or a maximum standardised uptake value of at least 12 [42]. Increasing PRIMARY Scores were associated with a greater probability of clinically significant disease. Unlike an isolated SUV threshold, this approach incorporates lesion location and uptake pattern and may reduce false-positive interpretation of diffuse activity in benign transition-zone tissue. External assessment has shown promising reproducibility, although broader validation is required before the score can be adopted as a routine equivalent to PI-RADS.

More recently, the PRIMARY2 trial extended this concept to men with equivocal or clinically high-risk non-suspicious MRI findings. In this multicentre phase III randomised non-inferiority trial, 660 men with PI-RADS 2 or 3 findings were assigned either to systematic transperineal biopsy or to a ^68Ga-PSMA-11 PET/CT-guided diagnostic pathway. The co-primary csPCa endpoint was Gleason 3 + 4 with at least 10% pattern 4 or higher, not unrestricted Grade Group ≥ 2. Detection was 12% in the PSMA PET/CT arm and 16% in the systematic-biopsy arm (difference −3.7%, 95% CI −8.9 to 1.5), meeting the prespecified 10% non-inferiority margin. Using the stricter threshold of Gleason ≥4 + 3, detection was 4.2% versus 4.8% (difference −0.63%, 95% CI −3.8 to 2.5). PSMA PET/CT allowed biopsy to be avoided in 49% of patients assigned to the experimental pathway. These findings provide prospective evidence that PSMA PET/CT may have a clinically relevant triage role in selected men with negative or equivocal MRI, reducing biopsy exposure without a clinically unacceptable loss in significant cancer detection [43].

The PEDAL study provided a direct prospective comparison between ^18F-DCFPyL PSMA PET/CT and mpMRI in men undergoing investigation for suspected prostate cancer. mpMRI showed greater diagnostic accuracy than PSMA PET as a stand-alone test, but the two modalities identified partly different lesions, and combined imaging reached a sensitivity of 98.8% with a negative predictive value of 96% [44]. This finding is consistent with their different biological targets: mpMRI assesses tissue architecture, diffusion, and perfusion, whereas PSMA PET reflects cell-surface target expression. A lesion inconspicuous on one modality can remain detectable on the other.

PSMA PET findings can be transferred to ultrasound for targeted biopsy. A meta-analysis of five prospective studies involving 497 men reported pooled sensitivity of 0.89 and specificity of 0.56 for clinically significant cancer [45]. Combined PSMA PET and MRI targeting increased pooled sensitivity to 0.91, with a negative predictive value of 0.85. A larger meta-analysis of 10 studies and 874 patients reported sensitivity of 0.89, specificity of 0.65, and an area under the summary receiver operating characteristic curve of 0.86 [46]. The evidence supports PSMA PET as a targeting adjunct, particularly for PI-RADS 3 lesions, but remains heterogeneous and is derived mainly from specialist centres [46].

Broader pooled analyses support the diagnostic value of PSMA PET, particularly when it is combined with mpMRI [47], and confirm a complementary role while showing that a negative PSMA PET examination cannot reliably exclude clinically significant disease [48]. Integration may improve triage in selected men with persistent suspicion after negative or equivocal MRI, but concordantly negative imaging should not independently justify biopsy omission.

Several limitations restrict routine pre-biopsy use. PSMA expression is heterogeneous, and some clinically significant cancers demonstrate low tracer uptake. Benign prostatic hyperplasia, prostatitis, and other non-malignant processes may also produce uptake, limiting specificity. Diagnostic thresholds, radiotracers, acquisition protocols, reporting systems, and biopsy reference standards remain inconsistent. PSMA PET also involves radiation exposure, higher cost, and more limited availability than ultrasound or MRI. Most published studies were performed in specialist centres and often retained systematic biopsy, preventing firm conclusions about whether molecular imaging can safely reduce background sampling.

PSMA PET is best regarded as an emerging problem-solving modality complementary to mpMRI, not a routine replacement for it. Its most plausible near-term role is in patients with equivocal or discordant findings, particularly when clinical suspicion remains high despite non-diagnostic MRI. Whether it can support the safe reduction in systematic biopsy will require prospective trials that directly compare complete and limited sampling strategies within clearly defined imaging-risk groups.

## 8. Toward an Integrated, Risk-Adapted Biopsy Strategy

No single biopsy template is appropriate for every man with suspected prostate cancer. Targeted-only biopsy may miss clinically significant disease because of MRI invisibility, lesion underestimation, targeting error, or multifocality, whereas a complete bilateral systematic template adds cores and increases low-grade cancer detection. Contemporary pathways should individualise the extent of background sampling.

The extent of additional sampling should reflect both the reliability of imaging and the patient’s residual risk. Full systematic biopsy remains reasonable when the probability of disease outside the MRI target is substantial. This may include patients with discordantly high prostate-specific antigen density, abnormal digital rectal examination, strong family history, previous atypical histology, multiple or bilateral MRI lesions, and cases in which imaging quality or targeting accuracy is uncertain. Broader sampling may also be justified in large glands, anteriorly located lesions, technically difficult biopsies, and settings where MRI interpretation or biopsy guidance is performed outside experienced centres. In such circumstances, the potential harm of missing or undergrading clinically significant cancer may outweigh the disadvantage of additional cores [2,4,5].

Reduced sampling is most plausible when the MRI lesion is focal and unilateral, imaging quality is satisfactory, and clinical risk is concordant. Most clinically significant cancers detected beyond targeted cores in the relevant studies were near the MRI-visible lesion or within the same lobe [20,21,24,27]. This concentration suggests that adjacent or ipsilateral cores can retain much of the diagnostic contribution of systematic sampling while avoiding low-yield contralateral biopsy. A narrow perilesional margin is more feasible with fusion or in-bore guidance, whereas ipsilateral systematic cores may provide a practical safety margin during cognitive targeting. Across the reduced-template studies reviewed here, omitting distant or contralateral cores missed approximately 0.3–3.2% of clinically significant cancers identified by the full combined approach, while saving a mean of five to six cores and reducing Grade Group 1 diagnoses. The acceptability of this trade-off depends on the individual patient’s residual risk.

Micro-ultrasound may further refine this pathway by providing real-time information during needle placement. A suspicious region that is concordant on mpMRI and micro-ultrasound could be sampled with greater confidence, while discordant findings may identify areas requiring additional cores. The OPTIMUM trial showed that micro-ultrasound-guided biopsy was non-inferior to MRI fusion-guided biopsy for the detection of Grade Group 2 or higher cancer, with systematic sampling retained in both arms, supporting its use as an alternative image-guided platform [40]. Evidence remains insufficient to assume that a negative micro-ultrasound examination can independently justify omission of background sampling.

PSMA PET may have a more selective role. Its principal value is likely to be in men with equivocal or negative MRI despite persistent clinical suspicion, or in cases with discordant clinical and imaging findings. In the PRIMARY study, the addition of PSMA PET to mpMRI increased sensitivity and negative predictive value for clinically significant disease [41]. Combined molecular and anatomical imaging may identify patients in whom biopsy can be more accurately directed, although cost, availability, radiation exposure, and variable PSMA expression currently limit routine pre-biopsy use.

An integrated strategy should combine clinical risk, imaging confidence, and procedural confidence. Clinical risk includes PSA density, digital rectal examination, family history, age, and previous biopsy results. The EAU matrix links PI-RADS and PSA density: for PI-RADS 1–2, biopsy is generally omitted below 0.20 ng/mL/cc and considered above this threshold; for PI-RADS 3, biopsy is omitted below 0.10, considered at 0.10–0.15, strongly considered at 0.15–0.20, and performed above 0.20 ng/mL/cc [15]. These thresholds inform rather than replace the broader clinical assessment. Imaging confidence reflects lesion conspicuity, location, concordance across modalities, and technical quality, which can be assessed systematically with PI-QUAL version 2 [49]. Procedural confidence includes operator experience, targeting method, and expected needle-placement accuracy. Together, these domains should guide selection among targeted-only, targeted plus perilesional or ipsilateral, and targeted plus full systematic biopsy.

**Figure 2:**
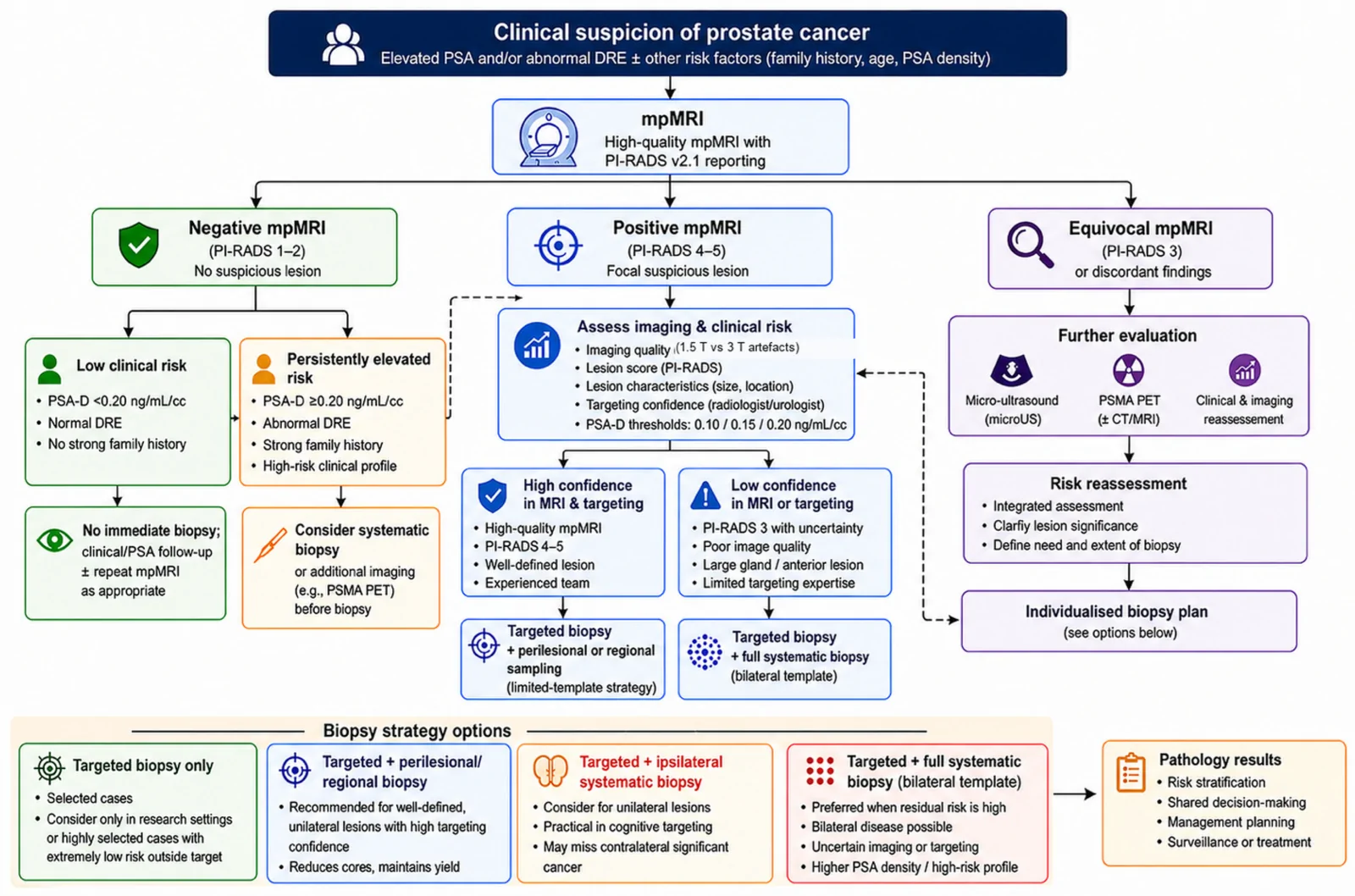
Proposed risk-adapted biopsy pathway in suspected prostate cancer. The pathway integrates pre-biopsy mpMRI, clinical suspicion, PSA density, MRI quality, lesion characteristics, and targeting confidence to determine whether biopsy can be deferred and, when indicated, the extent of sampling. After negative MRI (PI-RADS 1–2), biopsy may be omitted with PSA monitoring when clinical suspicion is low (PSA-D < 0.20 ng/mL/cc and no family history); persistent high suspicion supports systematic biopsy. After PI-RADS 3, biopsy may be omitted only when suspicion is very low (PSA-D < 0.10 ng/mL/cc and no family history); otherwise, targeted biopsy with perilesional sampling should be considered. PI-RADS 4–5 lesions should undergo targeted biopsy with perilesional sampling. Additional regional, ipsilateral, or full systematic sampling should be individualised according to residual risk, imaging quality, lesion distribution, and targeting confidence. MicroUS and PSMA PET may refine selected problem-solving cases but are not routine substitutes for the MRI-directed pathway. This conceptual framework is informed by EAU 2026 recommendations; it has not been prospectively validated and does not represent a guideline-endorsed standard of care. Abbreviations: DRE, digital rectal examination; EAU, European Association of Urology; mpMRI, multiparametric magnetic resonance imaging; MRI, magnetic resonance imaging; microUS, micro-ultrasound; PET, positron emission tomography; PI-RADS, Prostate Imaging Reporting and Data System; PSA, prostate-specific antigen; PSA-D, prostate-specific antigen density; PSMA, prostate-specific membrane antigen.

This framework is not a validated clinical algorithm and does not represent a guideline-endorsed standard of care. Evidence for reduced sampling is predominantly retrospective, definitions remain inconsistent, and direct comparisons of risk-adapted pathways are limited. Prospective trials should test predefined selection criteria rather than core counts alone, with preservation of clinically significant cancer detection and grading as the principal safety outcome. Figure 2 illustrates the proposed integration of clinical risk, mpMRI, imaging confidence, and biopsy extent.

## 9. Artificial Intelligence, Radiomics, and Future Directions

Artificial intelligence (AI) and radiomics may further refine prostate biopsy by extracting quantitative information that is not consistently captured through visual MRI interpretation alone. Radiomics converts imaging data into measurable features describing lesion intensity, shape, texture, and spatial heterogeneity. Machine-learning and deep-learning models can then combine these features with clinical variables such as prostate-specific antigen density, age, prostate volume, and PI-RADS score to estimate the probability of clinically significant prostate cancer [50,51,52].

The most immediate role of AI is decision support rather than autonomous diagnosis. It may assist lesion detection, reduce inter-reader variability, and improve assessment of equivocal findings, particularly PI-RADS 3 lesions. Multicentre studies report performance comparable to expert MRI interpretation, and combined imaging-clinical models may perform better than either data source alone [51,52,53]. In a prospective bicentre study, AI-assisted interpretation improved specificity while preserving high sensitivity, suggesting a potential reduction in false-positive MRI assessments [54].

Radiomics may also contribute to biopsy planning by estimating lesion aggressiveness before tissue sampling. Pooled evidence indicates high discriminatory performance for predicting Grade Group 2 or higher disease, although reported accuracy varies according to MRI sequences, segmentation method, reference standard, and validation strategy [55]. In principle, a high-risk quantitative signature could support more extensive targeted or regional sampling, whereas a low-risk and imaging-concordant lesion might justify a reduced-core approach.

Clinical implementation remains limited by retrospective, institution-specific models trained on relatively homogeneous datasets. Performance may decline across scanners, acquisition protocols, populations, and radiological workflows; manual segmentation and limited interpretability add further variability. External validation, calibration, prospective impact studies, and comparison with experienced radiologists are essential. PI-RADS Steering Committee reporting requirements reflect the need for transparent methods and clinically meaningful validation [56].

AI is best considered a risk-integration aid, not a replacement for radiologists or biopsy. Its clinical value depends on prospective evidence that it improves patient selection and safely reduces unnecessary cores without lowering clinically significant cancer detection. No published study discussed here tested an AI-defined reduction in core number; the PRIMER and AI-directed biopsy trials are among the first to address this question prospectively. Table 2 summarises representative evidence for established and emerging imaging-guided approaches.

## 10. Evidence Gaps and Priorities for Future Research

Evidence remains insufficient to define a universally applicable limited-biopsy protocol because most perilesional, regional, and ipsilateral studies retrospectively reconstruct a reduced strategy from cores obtained during a complete combined biopsy [27,30,31]. Such analyses do not fully reproduce a prospectively planned limited procedure.

Terminology and methods also vary. Perilesional sampling may denote a fixed distance from the MRI target, adjacent sectors, or the entire ipsilateral lobe; studies further differ in biopsy route, targeting technique, core number, previous biopsy status, lesion characteristics, MRI quality, and definitions of clinically significant cancer. This heterogeneity limits comparison and prevents identification of an optimal safety margin [21,30,31].

Another unresolved issue is patient selection. The diagnostic consequences of reducing systematic biopsy may differ according to PSA density, PI-RADS category, lesion size and location, prostate volume, MRI quality, targeting confidence, and the presence of unilateral or multifocal disease. Prospective trials should evaluate predefined risk-adapted strategies instead of applying one reduced template to all MRI-positive patients.

Emerging modalities require the same scrutiny. MicroUS and PSMA PET show promising diagnostic performance, but systematic cores were retained in many studies, obscuring their independent contribution [40,41]. AI models likewise require external validation across scanners, institutions, and populations before they can guide biopsy extent. Another question is whether improved guidance accuracy with mechanical or robotic guidance could support further reduction in systematic sampling. Other emerging imaging approaches, including optoacoustic imaging, optical coherence tomography, fluorescence-based techniques, and hybrid optical–MRI methods, are also being investigated for lesion characterisation or biopsy guidance. At present, however, their clinical role continues to be uncertain [57,58].

Future research should prioritise multicentre prospective comparisons of targeted-only, targeted-plus-regional, and targeted-plus-systematic strategies using standardised definitions. Outcomes should include upgrading at prostatectomy, repeat biopsy, complications, procedure duration, cost-effectiveness, patient-reported outcomes, and long-term oncological safety, not cancer detection alone. Ongoing trials are evaluating perilesional and regional sampling, multimodal imaging, AI-assisted targeting, and risk-adapted pathways (Table 3).

This review is narrative. Although a structured search and thematic selection criteria were used, there was no prospectively registered systematic-review protocol or formal risk-of-bias assessment. Study-selection and publication bias remain possible. In addition, the search for ongoing studies was limited to ClinicalTrials.gov, and trials registered exclusively in other international registries may therefore have been missed. Reference-list screening and prioritisation of prospective, multicentre, randomised, and pooled evidence reduced but did not eliminate this risk. The conclusions represent a critical narrative synthesis, not quantitative estimates from a formal systematic review.

## 11. Conclusions

Modern imaging has transformed prostate biopsy from a largely systematic procedure into a lesion-directed diagnostic pathway. Multiparametric MRI remains the central imaging modality, improving the detection of clinically significant prostate cancer while reducing unnecessary diagnosis of low-grade disease. Nevertheless, targeted biopsy alone does not eliminate the risk of missed or undergraded cancer, and additional sampling retains diagnostic value in selected patients.

The available evidence increasingly supports selective reduction, not universal omission of systematic biopsy. Perilesional, regional, and ipsilateral strategies may preserve most of the benefit of additional sampling while limiting unnecessary cores and clinically insignificant cancer detection. Micro-ultrasound and PSMA PET may further improve lesion localisation, whereas artificial intelligence could support risk integration and biopsy planning.

No single template is likely to suit every patient. Biopsy extent should be individualised according to clinical risk, imaging quality, lesion characteristics, and targeting confidence. Prospective multicentre validation remains necessary before reduced-sampling pathways can be adopted broadly.

## Figures and Tables

**Table 1:** Key studies evaluating reduced systematic sampling in MRI-positive patients.

Study	Design/Population	Targeting	Comparison	csPCa Detection/Consequence of Reduced Sampling	Key Limitation
Rouvière et al. (MRI-FIRST), 2019 [4].	Prospective multicentre paired diagnostic study; 251 biopsy-naïve men	MRI-targeted biopsy + SBx	TBx vs. SBx vs. combined biopsy	13/94 (13.8%) csPCa cases were detected only by SBx. Omitting SBx would have missed csPCa in 5.2% of the overall cohort.	Study evaluated complete omission rather than selective reduction in systematic sampling
Elkhoury et al. (PAIREDCAP), 2019 [17].	Prospective paired-cohort study; 248 biopsy-naïve men with MRI-visible lesions	Cognitive and software MRI/US fusion TBx	Targeted approaches vs. SBx vs. combined biopsy	Combined biopsy increased csPCa detection from 154/248 (62%) with targeted biopsy to 174/248 (70%); 20 additional csPCa cases were identified by adding systematic sampling	Single academic centre; evaluated omission rather than selective reduction in SBx
Bourgeno et al., 2024 [19].	Multicentre cohort; 2387 men; PI-RADS ≥ 3; 15 European centres	MRI-TBx + side-specific SBx	TBx + ipsilateral SBx vs. TBx + bilateral SBx	Only 35 patients (1.5%) had csPCa exclusively on contralateral SBx; the added csPCa detection from ipsilateral and contralateral SBx was 4.2% and 2.8%, respectively	Retrospective analysis; heterogeneous centre protocols; modest model performance (AUC 0.63)
Brisbane et al., 2022 [20].	Retrospective spatial analysis of prospective data; 2048 men; PI-RADS 3–5 lesions	MRI/US fusion TBx + SBx	Cores ≤ 10 mm vs. >10 mm from the nearest MRI lesion	90% of csPCa-positive cores were within 10 mm of the nearest MRI lesion; 26% were outside the ROI but within the 10 mm perilesional “penumbra”	Spatial mapping, not a prospective reduced-biopsy protocol; dependent on MRI/fusion accuracy
Noujeim et al., 2023 [21].	Single-centre consecutive cohort; 505 men; PI-RADS 3–5	MRI-TBx + 3D core mapping	TBx + 10 mm perilesional sampling vs. TBx + full SBx	csPCa detection was 35% with perilesional sampling versus 37% with full combined biopsy (absolute difference −2 percentage points; *p* = 0.20)	Observational; reconstructed reduced strategy; no external validation
Bryk et al., 2017 [22].	Single-centre cohort; 211 men; single unilateral MRI lesion	MRI/US fusion TBx	TBx + ipsilateral vs. bilateral SBx	TBx + ipsilateral SBx retained 96.0% of csPCa detected by the complete strategy while reducing ciPCa detection	Retrospective hypothetical recombination of full-biopsy cores
Freifeld et al., 2019 [23].	Retrospective cohort; 116 men; single MRI lesion	MRI/TRUS fusion TBx	TBx + ipsilateral vs. full SBx	Sensitivity for csPCa increased from 85.5% with TBx alone to 96.4% with TBx + ipsilateral SBx	Small single-centre cohort; hypothetical reduced strategy
Jager et al., 2022 [24].	Retrospective subgroup of a prospective 2-centre database; 228 men; unilateral PI-RADS ≥ 3	MRI-TBx + SBx	TBx + ipsilateral vs. bilateral SBx	TBx + ipsilateral SBx retained 98.6% of csPCa; only 1.4% of csPCa was missed while ciPCa detection was reduced by 15.6%	Mixed population; retrospective comparison
Ruan et al., 2023 [25].	Retrospective cohort; 464 men; MRI-visible PI-RADS ≥ 3 lesions	MRI-TBx (≥2 cores) + SBx	TBx + ipsilateral vs. full SBx	TBx + ipsilateral SBx would have missed only 0.3% of csPCa detected by the full combined approach	Reconstructed strategy; only 48 men had multiple lesions
Yu et al., 2024 [26].	Retrospective single-centre cohort; 180 men (122 with unilateral lesions)	Transrectal cognitive TBx	TBx + ipsilateral vs. full SBx	In unilateral lesions, TBx + ipsilateral SBx retained 95.9% of csPCa detected by the full approach	Small subgroup; no prostatectomy reference
Hagens et al., 2022 [27].	Retrospective analysis of prospective data; 235 biopsy-naïve men; unilateral PI-RADS ≥ 3	Transperineal cognitive TBx	TBx + perilesional vs. full SBx	Reduced sampling detected 92/95 (96.8%) csPCa cases; 3/95 (3.2%) would not have been detected by the reduced strategy	Reconstructed strategy; prospective validation required
Duwe et al., 2024 [28].	Prospective non-randomised, blinded, single-centre study; 190 men; 218 lesions	MRI/US fusion; 3–5 TBx + 6 perilesional cores	TBx + perilesional vs. TBx + 12-core SBx	csPCa detection was 30.7% with TBx + perilesional biopsy versus 39.9% with TBx + SBx (absolute difference −9.2 percentage points)	Single-centre; non-randomised; lesion-level analysis
Deng et al. (SEXTANT-PRO), 2025 [18].	Single-centre non-inferiority RCT; 506 biopsy-naïve men; solitary PI-RADS ≥ 3 lesion	Transperineal cognitive TBx	TBx + 6-core vs. 12-core SBx	csPCa detection was 54.3% versus 54.8% (absolute difference −0.5 percentage points); the 6-core strategy met the prespecified criterion for non-inferiority	Selected population; single centre; wide non-inferiority margin; uncertain generalisability
Jiang et al., 2024 [29]	Single-centre RCT; 434 biopsy-naïve men; PSA 4–20 ng/mL	MRI-directed regional saturation biopsy	9-core regional saturation vs. TBx + SBx	csPCa detection was 44.1% with regional biopsy versus 40.7% with TBx + SBx (*p* = 0.30), with fewer cores	Restricted PSA range; nonstandard 9-core template
Hagens et al., 2022 [30].	Systematic review/meta-analysis; 8 studies; 2603 MRI-positive men	Mostly MRI/TRUS fusion	TBx + regional vs. TBx alone or TBx + full SBx	No significant difference in GG ≥2 detection between regional and full systematic sampling (RR 0.95, 95% CI 0.90–1.01); regional biopsy detected more csPCa than TBx alone	Heterogeneous definitions; 5/8 studies retrospective; not formal equivalence
Sanguedolce et al., 2025 [31].	Systematic review/meta-analysis; 21 studies; 11,784 patients	Mixed MRI-targeting methods	TBx + regional vs. TBx + full SBx	csPCa detection was 44.2% with regional sampling versus 46.1% with full SBx (absolute difference −1.9 percentage points; *p* = 0.07); full SBx detected more GG1 disease	17/21 studies retrospective; marked methodological heterogeneity

Abbreviations: AUC, area under the curve; CI, confidence interval; csPCa, clinically significant prostate cancer; GG, Grade Group; MRI, magnetic resonance imaging; PI-RADS, Prostate Imaging Reporting and Data System; ROI, region of interest; SBx, systematic biopsy; TBx, targeted biopsy; TRUS, transrectal ultrasound; US, ultrasound.

**Table 2:** Key studies evaluating contemporary and emerging imaging-guided strategies for prostate cancer diagnosis and biopsy.

Study	Modality	Design/Population	Strategy	Main Result	Key Limitation
Ahmed et al. (PROMIS), 2017 [8].	mpMRI	Prospective paired study; 576 men; mpMRI, TRUS biopsy and template mapping	mpMRI as pre-biopsy triage	Sensitivity 93%, NPV 89%; 27% could avoid immediate biopsy	Historical csPCa definition; MRI did not direct biopsy
Kasivisvanathan et al. (PRECISION), 2018 [1].	mpMRI	Multicentre RCT; 500 biopsy-naïve men	MRI ± targeted biopsy vs. standard SBx	csPCa 38% vs. 26%; 28% avoided biopsy; fewer insignificant cancers	Negative-MRI arm not biopsied; MRI-occult cancer not fully assessed
van der Leest et al., 2019 [11].	mpMRI	Prospective multicentre comparative study; 626 biopsy-naïve men	MRI pathway with in-bore TBx vs. systematic TRUS biopsy	csPCa 25% vs. 23%; insignificant cancer 14% vs. 25%; 49% MRI-negative	High-quality expert MRI; generalisability uncertain
Klotz et al., 2021 [12].	mpMRI	Phase III RCT; 453 biopsy-naïve men	MRI ± TBx vs. systematic TRUS biopsy	GG ≥ 2: 35% vs. 30%; 37% avoided biopsy; GG1: 10% vs. 22%	MRI-negative men were not biopsied
Klotz et al., 2021 [37].	Micro-ultrasound	Prospective registry; 1040 men; 11 centres	microUS-directed vs. mpMRI-directed assessment, with background sampling	GG ≥ 2 sensitivity: 94% vs. 90%; NPV: 85% vs. 77%	Heterogeneous protocols; learning curve; incomplete blinding
Kinnaird et al. (OPTIMUM), 2025 [40].	Micro-ultrasound	Multicentre non-inferiority RCT; 802 randomised, 678 biopsied	microUS-guided vs. MRI/US fusion-guided biopsy; systematic cores in all	GG ≥ 2: 47.1% vs. 42.6%; microUS was non-inferior	Does not evaluate omission of systematic/background cores
Emmett et al. (PRIMARY), 2021 [41].	PSMA PET + mpMRI	Prospective multicentre study; 291 biopsy-naïve men	68Ga-PSMA PET/CT added to mpMRI before TP biopsy	Sensitivity: 83%→97%; NPV: 72%→91%	Specialist centres; cost/radiation; routine generalisability uncertain
Wong et al. (PEDAL), 2024 [44].	PSMA PET + mpMRI	Prospective multicentre paired study; 236 imaged, 184 biopsied	18F-DCFPyL PET/CT vs. mpMRI	mpMRI was more accurate alone; combined sensitivity 98.8%, NPV 96%	Not all participants biopsied; PSMA PET did not outperform mpMRI
Chow et al., 2025 [47].	PSMA PET + mpMRI	Systematic review/meta-analysis; 20 studies; 2153 patients	Combined PSMA PET + mpMRI vs. either modality alone	Combined sensitivity 92%, specificity 64%; better discrimination than mpMRI alone	Heterogeneous tracers, protocols, populations and reference standards
Buteau et al. (PRIMARY2), 2026 [43].	PSMA PET/CT	Multicentre phase III non-inferiority RCT; 660 men; PI-RADS 2–3	68Ga-PSMA-11 PET/CT-guided pathway vs. systematic TP biopsy	csPCa 12% vs. 16% (difference −3.7%, 95% CI −8.9 to 1.5); 49% avoided biopsy; non-inferior	Selected cohort; primary csPCa definition (Gleason 3 + 4 with ≥10% pattern 4) and 10% non-inferiority margin limit generalisation
Lee et al., 2025 [54].	AI/deep-learning MRI	Prospective bicentre study; 205 men; 259 lesions	Deep-learning model added to radiologist PI-RADS assessment	Specificity rose from 21%→44% while high sensitivity was maintained	Two-centre validation; external prospective testing needed
Lomer et al., 2025 [55].	MRI radiomics	Systematic review/meta-analysis; 43 studies; 9983 patients	MRI radiomics for GG prediction	Patient-level AUC 0.93 for GG ≥2	High methodological heterogeneity; variable segmentation and validation

Abbreviations: AI, artificial intelligence; AUC, area under the curve; csPCa, clinically significant prostate cancer; GG, Grade Group; microUS, micro-ultrasound; mpMRI, multiparametric magnetic resonance imaging; MRI, magnetic resonance imaging; NPV, negative predictive value; PET/CT, positron emission tomography/computed tomography; PI-RADS, Prostate Imaging Reporting and Data System; PSMA, prostate-specific membrane antigen; TRUS, transrectal ultrasound; US, ultrasound.

**Table 3:** Selected ongoing and planned clinical trials evaluating imaging-guided and risk-adapted prostate biopsy strategies.

Trial/ClinicalTrials.gov ID	Design/Population	Investigational Strategy	Comparator/Reference	N	Status	Primary Completion
PB-01/NCT06482645	RCT; biopsy-naïve men; solitary PI-RADS 4–5 lesion	TBx + perilesional biopsy (10–15 mm)	TBx + 12-core SBx	640	Recruiting	September 2026
TARGET/NCT07296042	Within-patient study; PI-RADS 4–5 or PI-RADS 3/PSA-D ≥0.15	TBx + 4 regional cores	Systematic cores reconstructed from the same biopsy	456	Not yet recruiting	March 2028
PRORAND/NCT07422597	Non-inferiority study; men with suspected PCa	ERSPC risk-calculator triage	Upfront MRI pathway	1016	Recruiting	December 2027
GUIDE PSMA PET/MRI/NCT07258056	Single-group study; PI-RADS 1–2 or negative MRI-target biopsy; PSA-D > 0.20	^18^F-PSMA-1007 PET/MRI-targeted biopsy	Systematic biopsy in the same patients	100	Recruiting	December 2028
PSMA PET/MRI-guided biopsy/NCT06923657	RCT; previous negative biopsy; PI-RADS ≤ 3; persistent PSA/PHI elevation	PSMA PET/MRI-TRUS-targeted biopsy	Transperineal saturation biopsy	230	Recruiting	September 2026
BiPASS/NCT07052214	Phase III single-arm study; biopsy-naïve men	PSMA PET + MRI ± targeted biopsy	Standard template biopsy in all patients	204	Recruiting	June 2026
AI Algorithm-Informed Biopsy/NCT07231627	Phase I/II RCT; PI-RADS 3 or low-risk MRI/high-risk features	AI-directed TBx + perilesional biopsy	Contemporary biopsy pathway	50	Recruiting	January 2028
PRIMER/NCT07162194	Within-patient diagnostic trial; MRI/TRUS biopsy	Deep- and green-learning AI targets	Radiologist PI-RADS targets; systematic reference	130	Ongoing	October 2027
AI Impact on Prostate Cancer Workflow/NCT07084779	Interventional study; pre-biopsy MRI	Radiologist + AI; optional AI-only targets	Radiologist alone + systematic reference	150	Recruiting	November 2026

Abbreviations: ^18^F, fluorine-18; AI, artificial intelligence; ERSPC, European Randomised Study of Screening for Prostate Cancer; MRI, magnetic resonance imaging; N, planned sample size; PCa, prostate cancer; PET, positron emission tomography; PHI, Prostate Health Index; PI-RADS, Prostate Imaging Reporting and Data System; PSA, prostate-specific antigen; PSA-D, prostate-specific antigen density; PSMA, prostate-specific membrane antigen; RCT, randomised controlled trial; SBx, systematic biopsy; TBx, targeted biopsy; TRUS, transrectal ultrasonography. Trial status, planned enrolment, and estimated primary completion dates were obtained from ClinicalTrials.gov. Registry information may change over time.

## Data Availability

No new data were created or analysed in this study. Data sharing is not applicable to this article.
